# P-118. Does Antibiotic Therapy Affect Multiplex PCR Results in Community-Acquired Bacterial Meningitis?

**DOI:** 10.1093/ofid/ofaf695.346

**Published:** 2026-01-11

**Authors:** Caroline I Reckart, Elizabeth A Aguilera, Audrey Wanger, Rafael Bravo-Santos, Rodrigo Hasbun

**Affiliations:** McGovern Medical School at the University of Texas Health Sciences Center at Houston, Houston, TX; UTHealth, McGovern Medical School, Houston, Texas; McGovern Medical School, Houston, Texas; The University of Texas Health Sciences Center in Houston, Houston, Texas; UT Health Mc Govern Medical School, Houston, Texas

## Abstract

**Background:**

Community-acquired bacterial meningitis (CABM) can be microbiologically confirmed either by cerebrospinal fluid (CSF) culture, blood culture and/or by multiplex CSF PCR. Antibiotic therapy impacts the microbiologic yield and the CSF profile in bacterial meningitis but the impact on bacterial CSF PCR is not known. The goal of our study was to assess how timing of antibiotic administration and the delay in obtaining CSF collection impacted the microbiological yield of the aforementioned methods in patients with proven CABM.

**Figure:**
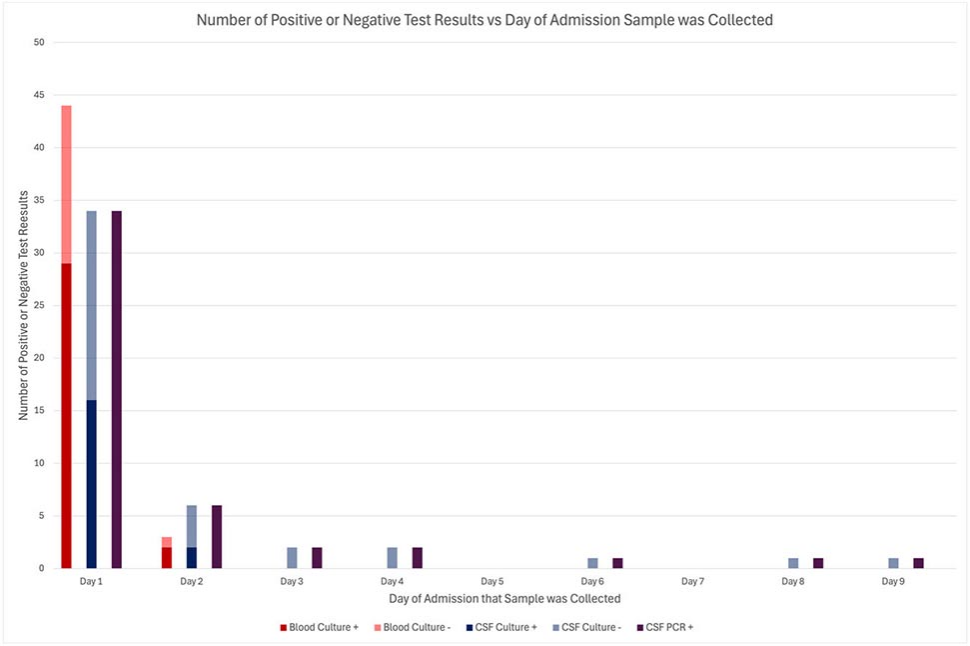

**Methods:**

A retrospective study of 490 adults and children with meningitis and encephalitis that underwent evaluation with the Biofire® multiplex PCR panel from June 2018 to August 2024. Patients were sourced from 15 hospitals in the Greater Houston area.

**Results:**

Out of 490 patients with meningitis and encephalitis, 47 (9.6%) had CABM: 47 (100%) were positive by CSF PCR; 32 (68.1%) had a positive blood culture and only 18 (38.3%) had a positive CSF culture. Two-thirds (61.7%) of patients received antibiotics before CSF was obtained while one-third (27.7%) of patients underwent lumbar puncture more than 24 hours after presentation to the emergency department. CSF PCR tested positive up to 9 days after presentation, even after the administration of antibiotics.

**Conclusion:**

In CABM, antibiotic therapy does not appear to impact the yield of the bacterial CSF PCR. In our study, 100% of patients had a positive CSF PCR result, even up to 9 days after initiation of antibiotic therapy.

**Disclosures:**

Rodrigo Hasbun, MD MPH FIDSA, Biomeriaux: Grant/Research Support|Biomeriaux: research funding and personal fees to help with Monte Carlo simulation studies evaluating impact of cost on adults and children

